# Paradoxical attenuation of neuroinflammatory response upon LPS challenge in miR-146b deficient mice

**DOI:** 10.3389/fimmu.2022.996415

**Published:** 2022-10-31

**Authors:** Keerthana Chithanathan, Monika Jürgenson, Mithu Guha, Ling Yan, Tamara Žarkovskaja, Martin Pook, Nathaniel Magilnick, Mark P. Boldin, Ana Rebane, Li Tian, Alexander Zharkovsky

**Affiliations:** ^1^ Department of Physiology, Institute of Biomedicine and Translational Medicine, Faculty of Medicine, University of Tartu, Tartu, Estonia; ^2^ Department of Pharmacology, Institute of Biomedicine and Translational Medicine, University of Tartu, Tartu, Estonia; ^3^ Department of Biomedicine, Institute of Biomedicine and Translational Medicine, University of Tartu, Tartu, Estonia; ^4^ Department of Molecular and Cellular Biology, Beckman Research Institute of City of Hope National Medical Center, Duarte, CA, United States

**Keywords:** miRNAs, miR-146b deficiency, attenuated neuroinflammation, miR-146a upregulation, miR-146a/b crosstalk

## Abstract

The miR-146 family consists of two microRNAs (miRNAs), miR-146a and miR-146b (miR-146a/b), both of which are known to suppress immune responses in a variety of conditions. Here, we studied how constitutive deficiency of miR-146b (*Mir146b-/-*) affects lipopolysaccharide (LPS)-induced neuroinflammation in mice. Our experiments demonstrated that miR-146b deficiency results in the attenuation of LPS-induced neuroinflammation, as it was evidenced by the reduction of sickness behavior, a decrease in the inflammatory status of microglia, and the loss of morphological signs of microglial activation in the hippocampus. Gene expression analysis revealed that LPS-induced upregulation of hippocampal pro-inflammatory cytokines is attenuated in *Mir146b-/-* mice, compared to wild-type (*WT*) mice. In addition, reduced expression of the NF-κB nuclear protein p65, reduced miR-146 family target TLR4 expression and relatively stronger upregulation of miR-*146a* was found in *Mir146b-/-* mice as compared to *WT* mice upon LPS challenge. Compensatory upregulation of miR-146a can explain the attenuation of the LPS-induced neuroinflammation. This was supported by experiments conducted with miR-146a/b deficient mice (*Mir146a/b-/-*), which demonstrated that additional deletion of the miR-146a led to the restoration of LPS-induced sickness behavior and proinflammatory cytokines. Our experiments also showed that the observed upregulation of miR-146a in *Mir146b-/-* mice is due to the overexpression of a miR-146a transcription inducer, interferon regulatory factor 7 (*Irf7*). Altogether, our results show the existence of crosstalk between miR-146a and mir-146b in the regulation of LPS-induced neuroinflammation.

## Introduction

Neuroinflammation is considered as an important contributing factor in various central nervous system disorders, including neuropsychiatric disorders and neurodegenerative diseases, such as major depression, schizophrenia, Alzheimer’s disease, and Parkinson’s disease (1–5). During inflammation, the termination phase is crucial to prevent tissue damage. For that, effective resolution programs are needed to prevent the progression of persistent inflammation (6). In this regard, several microRNAs (miRNAs) have been demonstrated to have a critical role in controlling inflammation (7). miRNAs are endogenous, small non-coding RNAs with ∼22 nucleotides in length, which regulate gene expression *via* imperfect base-pairing with the 3′-untranslated regions (3′-UTR) of multiple target mRNAs and thereby inhibit mRNA and protein expression (8). Individual miRNAs can affect different aspects of innate as well as adaptive immune cell development and functions (9). Several miRNAs have emerged as regulators of neuroinflammation, among which miR-155 has been shown to promote (10) and the miR-146 family to suppress neuroinflammation (11, 12).

The miR-146 family of miRNAs consists of two members, miR-146a and miR-146b (miR-146a/b), which differ only in two nucleotides and are encoded by two distinct genes located on chromosomes 5 and 10, in the human genome and on chromosome 11 and 19 in the mouse genome, respectively (13). As miR-146a/b have identical seed sequences, they can probably target the same or very similar set of genes (13, 14). Both miR-146a/b have been shown to negatively regulate the nuclear factor kappa B (NF-κB) pathway through multiple targets such as the TNF receptor-associated factor 6 (TRAF6) and interleukin-1 receptor-associated kinase 1 (IRAK1) (15). Correspondingly, miR-146a/b have been shown to suppress innate immune responses in cell culture conditions (16) and *in vivo* in mice, as demonstrated by the exaggerated inflammatory response of miR-146a knockout mice upon administration of lipopolysaccharide (LPS) (17, 18). The lack of miR-146a or miR-146a/b also leads to changes in adaptive immune responses, as more prominent T helper (Th)1/Th17 responses have been revealed in these mice in different disease models (19–21).

In the central nervous system, miR-146a is abundantly expressed in microglial cells (22, 23). Numerous lines of genetic evidence suggest that polymorphisms in miR-146a can influence its function and thereby contribute to susceptibility to several neurological diseases (24, 25). In addition, miR-146a is upregulated in age-related inflammatory neurodegenerative disorders, including Parkinson’s disease (26), Alzheimer’s disease (27), viral encephalitis (28), and epilepsy (29).

In contrast to miR-146a, very few studies have addressed miR-146b function in the regulation of neuroinflammation in the central nervous system. Overexpression of miR-146b was shown to inhibit inflammatory responses *via* suppression of the activation of the NF-κB signaling in the brain in rat encephalopathy models (30). Another study showed that treatment of microglial cells with LPS led to increased expression of miR-146b but not miR-146a, while transfection of miR-146b into microglial cells significantly decreased LPS-induced microglial activation and downregulated IRAK1 (31). Intriguingly, we reported that constitutive miR-146b deficiency didn’t cause microglial activation or signs of neuroinflammation in the hippocampus of the mouse brain (22). However, knowledge on the regulation of neuroinflammation in the absence of miR-146b is still lacking.

Hereby, we used *Mir146b-/-* mice to study the role of miR-146b in neuroinflammation upon LPS challenge. Interestingly, we observed blunted neuroinflammatory response in miR-146b deficient mice subjected to LPS treatment which may be due to the elevated miR-146a level, causing downregulation of inflammatory factors.

## Materials and methods

### Animals

Two-three months old male C57BL/6J *WT*, *Mir146b-/-* and *Mir146a/b-/-* were housed and bred in laboratory animal facility at the Institute of Biomedicine and Translational Medicine, University of Tartu under standard conditions as previously described in (14). *Mir146b-/-* and corresponding *WT* mice used for this study were obtained by crossing *Mir146b+/-* heterozygous mice. The *Mir146a/b-/-* mouse line was created by crossing *Mir146b-/-* and *Mir146a-/-* lines. The generated animals were genotyped using the following primer sequence: 146b locus 5′ forward primer- 5′ CTCACACTCTTGTTCTTACCCAGTTCTT 3′; 146b locus 3′ reverse primer- 5′ CAAACAAACAAACAAAAGGTTCAGCTAAG 3′; 146b locus internal reverse primer- 5′ACACACAGGGCATATGAGATCAGTTGGTT 3′; 146a forward primer- 5’ ACCAGCAGTCCTCTTGATGC 3’; 146a reverse primer- 3’ GACGAGCTGCTTCAAGTTCC 5’ and same generation littermates were used in experiments. All experiments were undertaken in agreement with the guidelines established in the Principles of Laboratory Animal Care (Directive 2010/63/EU) and the mice were group-housed with a 12h light/dark cycle with food and water available ad libitum. The Animal Experimentation Committee at the Estonian Ministry of Agriculture (no. 183 and 158, 2021) approved the experimental protocol.

### Experimental plan

**Figure 1:**
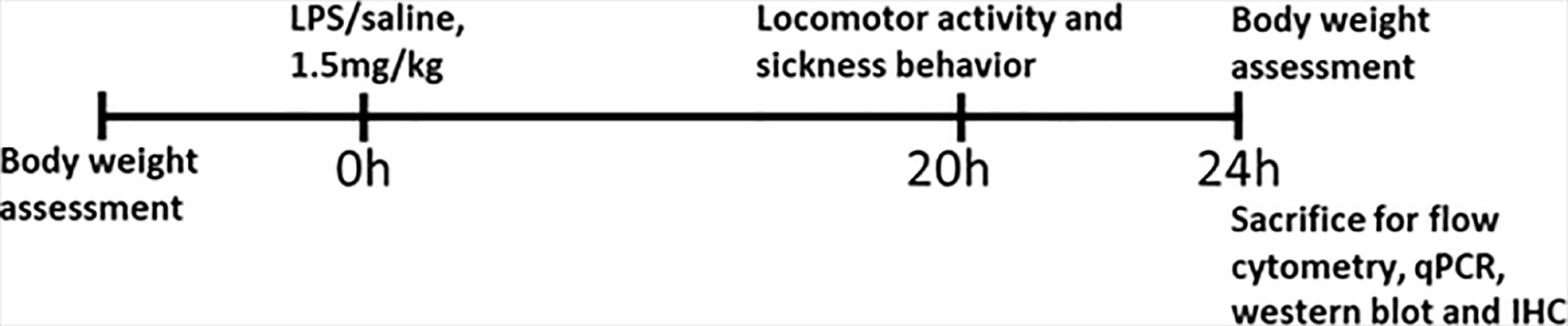
Schematic overview of the experimental plan.

Male *WT* (n = 40) and *Mir146b-/-* mice (n = 44), were randomly assigned to different experimental groups. The first cohort (*WT* n = 12, *Mir146b-/-* n = 13) was used to measure bodyweight, locomotor activity and sickness behavior after LPS or saline administration during the 20h period and animals were sacrificed at 24h. This cohort was used to determine LPS-induced genotypic differences in microglial profile by flow cytometry. The second cohort (*WT* n = 12, *Mir146b-/-* n = 13) was used for gene expression analysis, miRNA quantification and Western blot, besides behaviors. The third cohort (*WT* n = 10, *Mir146b-/-* n = 12) was used for morphological analysis of ionized calcium binding adaptor molecule 1 (Iba-1) positive cells by immunohistochemistry. Fourth cohort (*WT* n = 6, *Mir146b-/-* n = 6) was used for isolation of microglial cells after LPS administration. In addition, Male *WT* (n = 10) and *Mir146a/b-/-* mice (n = 10) was used to measure sickness behavior after LPS or saline administration followed by gene expression analysis. The sequence of the experiments performed is summarized in **Figure 1**.

### LPS treatment

LPS (derived from E. coli serotype O55:B5; Sigma-Aldrich, St. Louis, MO, USA) was dissolved in 0.9% NaCl (saline). In each animal cohort, one group was injected intraperitoneally (i.p.) with 1.5 mg/kg bodyweight of LPS solution and another group received injection of an equal volume of saline as controls. For isolated microglial cells from *WT* mice, 1 μg/mL of LPS for 4 h was used for stimulation.

### Body weight measurement and assessment of locomotor activity

Bodyweight was measured before and 24 h post LPS or saline injection in *WT* and *Mir146b-/-* mice. The effect of LPS on locomotor activity was monitored for 60 or 30 min by using PhenoTyper apparatus (Noldus, Leesburg, VA) with Ethovision XT live videotracking software (Version 8.0, Noldus Information Technologies, Leesburg, VA). Total distance traveled in the whole arena was measured.

### Tail suspension test

Tail suspension test was performed as previously described (32). In separate compartments of the wooden test apparatus, each mouse was suspended by the tail to a wooden beam with an adhesive tape. The total duration of immobility time during the 6 min testing period was measured manually by using stopwatch. Immobility was defined as a complete lack of movement other than respiration, small movements of forefeet and swinging caused by earlier movements.

### Flow cytometry

Flow cytometry was conducted according to the validated method published previously (33, 34). Mice were euthanized with CO2 and the brain tissues were dissected out. Isolation of cells were made by homogenizing the tissues through 70 µm cell strainers (#352350, BD Bioscience) in ice-cold flow buffer (PBS with 1% fetal calf serum). Cells were blocked with 10% rat serum in ice-cold PBS for 1 h and stained with 0.5 µl anti-mouse CD11b-Brilliant Violet 421 (cat no. 101251, Biolegend), CD45-Brilliant Violet 650 (cat no. 103151, Biolegend), MHCII-Brilliant Violet 711 (cat no. 107643, Biolegend), CD115-Brilliant Violet 605 (cat no. 135517, Biolegend), Glast-APC (cat no. 130-123-555, Miltenyi) with the corresponding isotype control antibodies (all from BioLegend) rat IgG2b-BV421 (#400639), rat IgG2b-BV650 (#400651), rat IgG2b-BV711 (#400653), rat IgG2a-BV605 (#400539) and mouse IgG2a-APC (#400219) in flow buffer for 1h. Cells were then fixed by 4% paraformaldehyde followed by permeabilization with 0.05% TritonX-100 in PBS at 4°C. After washings, cells were incubated with an anti-VGLUT2 mAb-Alexa488 (# MAB5504A4 Millipore) or isotype ctrl mAb-Alexa488 (#400132 Biolegend) for 1h at 4°C. Washed and resuspended cells were acquired with a Fortessa flow cytometer (BD Bioscience). Data were analyzed by Kaluza v2.1 software (Beckman Coulter). The cell viability was assessed using CytoTell orange (Cat. No. 22257, AAT Bioquest, US) and respective plots are shown in **Supplement Figure 2A**. Microglial cells were gating was based on the expression levels of CD11b and CD45 markers. Within the CD11b+CD45+ population, we gated CD11b+CD45intermediate as microglia and CD11b+CD45high as macrophages.

### Immunohistochemistry of Iba1

After 24 h of LPS administration, animals were deeply anesthetized with chloral hydrate (300 mg/kg, i.p.) and transcardially perfused using 0.9% saline and then with 4% paraformaldehyde (PFA) in phosphate buffered saline (PBS, pH = 7.4). The brains were kept in PFA solution for 72 h to reduce unspecific binding to endogenous biotin and 40 µm-thick sections were cut on a Leica VT1000S vibro-microtome (Leica Microsystems Pvt Ltd., Wetzlar, Germany) and stored at −20°C in the cryo-protectant (30% ethylene glycol, 30% glycerol in PBS: pH 7.4).

Before staining, the sections were washed three times in PBS and treated with 2% H2O2 solution for 20 min followed by incubation in 0.01 M citrate buffer (pH 6.0) at 85°C for 30 min in water bath and then stood for 30 min at room temperature. Another wash was done in PBS containing 0.1% Triton X-100. Blocking was done with solution containing 5% goat serum, 0.5% Tween-20, 0.25% Triton X-100 in 100 mM PBS for 1 h. Iba1 primary antibody (1: 700, rabbit anti-Iba1, CAF6806, FUJIFILM Wako Chemicals Europe GmbH, Neuss, Germany) was added in blocking buffer for 72 h. After being washed three times, the sections were incubated with secondary antibody (1:700, affinity purified goat anti-rabbit biotinylated IgG (H+L), Vector Laboratories) in blocking buffer at room temperature for 1 h. Iba1-positive cells were visualized using peroxidase method (ABC system and diaminobenzidine as chromogen, Vector Laboratories). The sections were dried, cleared with xylol and cover-slipped with mounting medium (Vector Laboratories, Newark, CA, USA). To control unspecific binding, some sections were used as negative controls, where incubation step with the primary antibody was omitted.

The morphological characteristics of Iba1+ microglia (cell size, cell body size, size dendritic processes), were analyzed using image analysis software (ImageJ 1.48v, http://imagej.nih.gov/ij), an algorithm previously described (35) was used. Briefly, images were converted into binarized 8-bit format and “adjusted threshold” and “analyze particles” functions were used to apply intensity thresholds and size filter. To measure the total cell size, the threshold was maintained at the level that was automatically provided by the ImageJ program, and size filter of 150 pixels was applied. To measure the total cell body size, the threshold was lowered by 40 points and no size filter was applied. The counts of Iba-1 positive cells were obtained from images according to the algorithm described previously (36) using “analyze particles” command in ImageJ software.

### Isolation of microglia

Brain cells were isolated as previously described (37). Tissues were mechanically homogenized and passed through a 70 μm nylon cell strainer (352350, BD Bioscience) with approximately 10–15 ml of 1X DPBS supplemented with 0.2% glucose into a 50 ml conical tube. Isotonic percoll dilutions were made by diluting stock percoll (GE-healthcare, 17-0891-01, Chicago, IL, USA) at a 9:1 ratio with 10X PBS to make stock isotonic percoll (SIP), which is considered 100% SIP. The layers of percoll were created by diluting the 100% SIP with 1X Dulbecco’s phosphate buffered saline (DPBS) to make 70% SIP, 50% SIP and 35% SIP. Obtained homogenate was then centrifuged at 600× g for 6 min at room temperature. Supernatant was decanted and the pellet resuspended in 6 ml of the 70% SIP. Resuspended homogenate was transferred to a 15 ml tube and 3 ml of the 50% SIP was carefully layered over. Another 3 ml of the 35% SIP was carefully layered on top of the 50% SIP layer, and 2 ml of 1X DPBS was layered on top of the 35% layer. The prepared 15ml tubes were then centrifuged at 2000× g for 20 min at room temperature without brake. Three discrete layers were established after centrifugation. Microglial cells were collected from the interface between the 50–70% SIP. Isolated microglial cells were resuspended in sterile 1X DPBS and centrifuged at 600× g for 6 min at room temperature to remove any remaining percoll. Washed cells were subjected to qPCR and flow cytometry methods to check purity of isolated microglial cells.

### Total RNA isolation, real-time quantitative PCR and absolute quantification by digital PCR

Total RNAs were extracted from brain tissues (hippocampus) by using TRI Reagent^®^ (TR 118) (Molecular Research Center, Inc., Cincinati, OH, USA). To measure mRNA expression, cDNA was synthesized using RevertAid First Strand cDNA Synthesis Kit (Thermo Fisher Scientific) followed by qPCR using 5 × HOT FIREPol^®^ EvaGreen^®^ qPCR Supermix (Solis BioDyne, Tartu, Estonia) on a QuantStudio 12KFlex instrument (Thermo Fisher Scientific) according to the instructions of the respective manufacturers. Primer sequences for target genes were given in the **Supplement Table 1**. Absolute quantification of microRNAs was carried out according to the manufacturer’s instructions (Qiagen) using miRCURY LNA RT Kit (Cat. No. 339340) for cDNA synthesis and miRCURY LNA PCR Assays (Assay IDs: YP00204688, YP02119310) in combination with QI Acuity EG PCR Kit (Cat. No. 250111) for detection of miR-146a and miR-146b. The samples were run and analyzed on QIAcuity One dPCR System using QIAcuity 26k Nanoplates (all from Qiagen). Relative miRNA expression was measured using TaqMan^®^ MicroRNA Assays hsa-miR-146a (Assay ID: 000468, Life technologies) and TaqMan^®^ MicroRNA Assays hsa-miR-146b (Assay ID: 001097, Life technologies, Carlsbad, CA, USA) according to the manufacturer’s instructions. For cRNA synthesis, TaqMan^®^ MicroRNA reverse transcription kit (4366596, Thermo Scientific) and for qPCR, 5× HOT FIREPol^®^ Probe qPCR Mix Plus (ROX) (Solis BioDyne) were used, respectively. U6 snRNA (Assay ID: 001973, Life Technologies) was used for the normalization of RT-qPCR. To analyze relative mRNA expression, ΔΔCt calculations was used. As housekeeping genes for normalization, *Gapdh* was used. The data were analyzed in relative to the mean value of one of the samples as a calibrator (control sample or WT saline), which was normalized to 1 and the rest of the samples were compared against the control sample.

### Western blot

Western blotting of Iba1 and toll-like receptor-4 (TLR4) protein was performed as described previously (38). Hippocampal tissues (15-20mg) were lysed in a lysis buffer (#ab113474; Abcam, UK) for protein extraction. The protein concentration was determined using the Bradford reagent kit (#B6916; Sigma-Aldrich, Germany). For Western blot analysis, 20 µg of total protein for TLR4 or Iba-1 detection was dissolved in 15% sodium dodecyl sulfate polyacrylamide gels and transferred electrophoretically onto a nitrocellulose membrane (Merck Millipore, US). The membranes were blocked with Intercept (TBS) Blocking Buffer (Li-Cor Biosciences, US) for 60 min at room temperature (RT). Then, the blots were incubated for 48h at 4°C with the primary antibody for Iba1 (Rb, #ab178846, 1:1000; Abcam, UK) or TLR4 (Ms, #sc-293072, 1:500; Santa Cruz Biotechnology, USA) and after washing thrice with TBST (50 mmol/L Tris, pH 7.6; 0.9% NaCl; and 0.1% Tween-20), the membranes were incubated with the secondary antibody (IRdye800, aRb, #C40721-02, 1:10000; Li-Cor Biosciences) for 1h at RT. Loading control β-actin was detected by incubating membranes overnight with monoclonal anti-β-actin antibody (Ms, #039M4768V, 1:10000; Sigma-Aldrich, US), followed by incubation with IRDye conjugated secondary antibody (IRdye680LT, aMs, #C60301-03, 1:10000; Li-Cor Biosciences). After extensive washing with TBST, the Odyssey Infrared Imaging System (Li-Cor Biosciences) was used for antibody detection and quantification. The ratios of Iba1 or TLR4 protein to β-actin were calculated and expressed as the mean optical density ratio in arbitrary units.

For NF-κB p65 protein in the nuclear fraction was performed as follows. Nuclear extract was prepared from the hippocampal tissues according to manufacturer’s protocol (Abcam nuclear extraction kit, ab113474) using 20 mg tissue. Protein content of nuclear fractions were measured using Bradford reagent (Sigma, B6916). Equivalent amounts of proteins were resolved on 10% polyacrylamide gels by SDS-PAGE. Resolved proteins were transferred to Immobilon^®^ FL membrane (IPFL00010, Millipore) in 0.1 M Tris-base, 0.192 M glycine and 20% (w⁄w) methanol using an electrophoretic transfer system with cold-block. The membranes were blocked using Odyssey blocking buffer (LICOR Biosciences, 927-40000) at room temperature for 1 h. After blocking, the membranes were incubated sequentially overnight with different primary antibodies anti rabbit NF-kB p65 (Abcam antibody, ab 16502), anti-rabbit histone-4 antibody to mark the nuclear fraction (Abcam antibody, ab 10158), anti-mouse beta-tubulin antibody to show no cytoplasmic contamination in nuclear fractions (Milipore, MAB1637). Incubations were followed by 3 times washing in Tris Buffered Saline containing 0.1% Tween-20 followed by incubation with the appropriate secondary antibody (1:10000), either goat anti-rabbit IRDye 800CW or 680LT or goat anti-mouse IRDye 680LT (all from Licor Biosciences), for 2 h at room temperature. Immuno-reactive bands were detected by the Odyssey Infrared Imaging System. Densitometry analysis of protein bands were done by Image studio Ver 2.0. Ink.

### Statistical analysis

GraphPad 8.0.1 (San Diego, CA, United States) was used for statistical analyses and graphical presentations. The following steps of statistical evaluation of the data were undertaken. All data sets were tested for homogeneity of variances using the Brown-Forsythe test and for normal distribution using the Shapiro-Wilk test. If the data sets passes both the tests then they were processed using two-way ANOVA followed by Tukey multiple comparisons test. If data sets did not pass a test for homogeneity of variances, they were processed using Welch’s ANOVA followed by Dunnett’s test. If the data did not pass the Shapiro-Wilk test, they were transformed into logarithms (Y=Log(Y)) and processed further using one-way ANOVA followed by Tukey multiple comparisons test. The appropriate statistical test for each data set is specified in the figure legends. Statistical significance was set at p < 0.05, and data were reported as mean ± SEM.

## Results

### Expression profile of miR-146a/b upon LPS in the hippocampus and microglial cells isolated from the mouse brain

**Figure 2:**
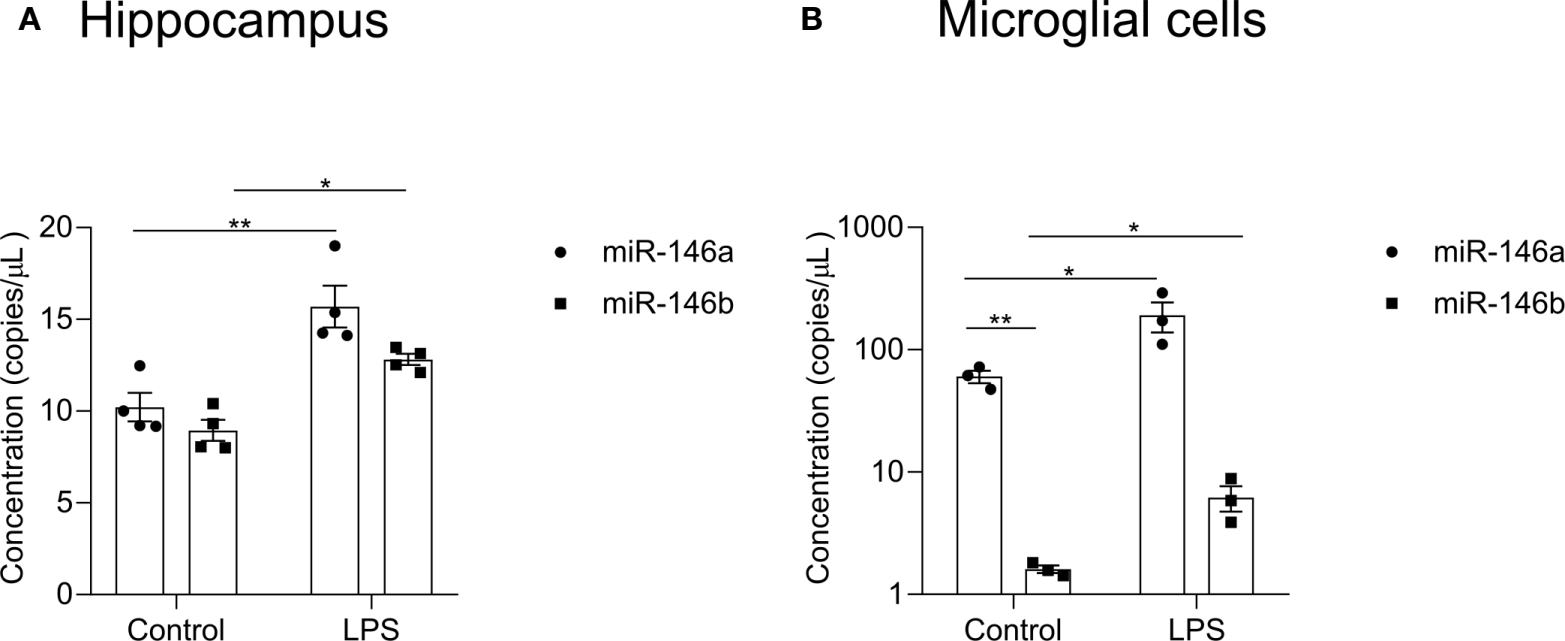
Expression of miR-146a/b in hippocampal tissue and microglia cells from *WT* mice. The expression analysis was performed in mice injected with LPS in saline or equal volume of saline (control). **(A, B)** miR-146a/b expression in the hippocampus and microglial cells. Number of animals = 3-4. Data are represented as mean ± SEM; * p < 0.05, ** p < 0.01 (Two-way ANOVA with Tukey’s multiple comparison test).

To better understand the importance of miR-146a/b in neuroinflammation, we first measured the expression levels of miR-146a/b in the hippocampus of *WT* mice upon LPS treatment using RT-qPCR method that enables absolute quantification. In hippocampal tissues, basal levels of miR-146a/b were similar, and after LPS administration both miR-146a (p = 0.0013) and miR-146b (p = 0.0170) were significantly induced at 24 h time point. The upregulation of miR-146a was apparently stronger, compared to miR-146b (p = 0.0836) (**Figure 2A**). Next, we evaluated expression of miR-146a/b in microglial cells isolated from the *WT* mouse brain. Purity of isolated microglia is shown in **Supplement Figure 1**. In microglial cells, the basal expression of miR-146a was several folds higher than that of miR-146b, while upon LPS challenge, both miR-146a (p = 0.0340) and miR-146b (p = 0.0342) were upregulated (**Figure 2B**). Significant effects of LPS treatment (F(1,8) = 6.475, p = 0.0345), miR expression (F(1,8) = 20.97, p = 0.0018), and interaction (miR expression × treatment: F(1,8) = 5.629, p = 0.0451) existed. Altogether, these data demonstrate that miR-146a was over 37-fold more highly expressed in microglial cells under basal conditions, and that both miR-146a/b were induced upon LPS treatment in the hippocampus and microglial cells of mouse brain. In contrast, the level of induction of miR-146b was little more prominent than miR-146a in microglial cells.

### LPS-induced reduced sickness behavior in *Mir146b-/-* mice

We have previously demonstrated that *Mir146b-/-* mice did not differ from *WT* animals with regard of body weight, locomotor activity, food and water consumption, hippocampal and total brain volumes at the age of 2-3 months (22). It is known that systemic administration of LPS in animals leads a behavioral state called “sickness behavior”, which is characterized by lethargy, decreased locomotor activity and appetite, depression-like behavior, and increased sensitivity to pain (39). To evaluate the impact of miR-146b on the LPS-induced sickness behavior, we treated *Mir146b-/-* and *WT* mice with LPS and assessed the locomotor activity, loss of body weight and immobility time in tail suspension test.

**Figure 3:**
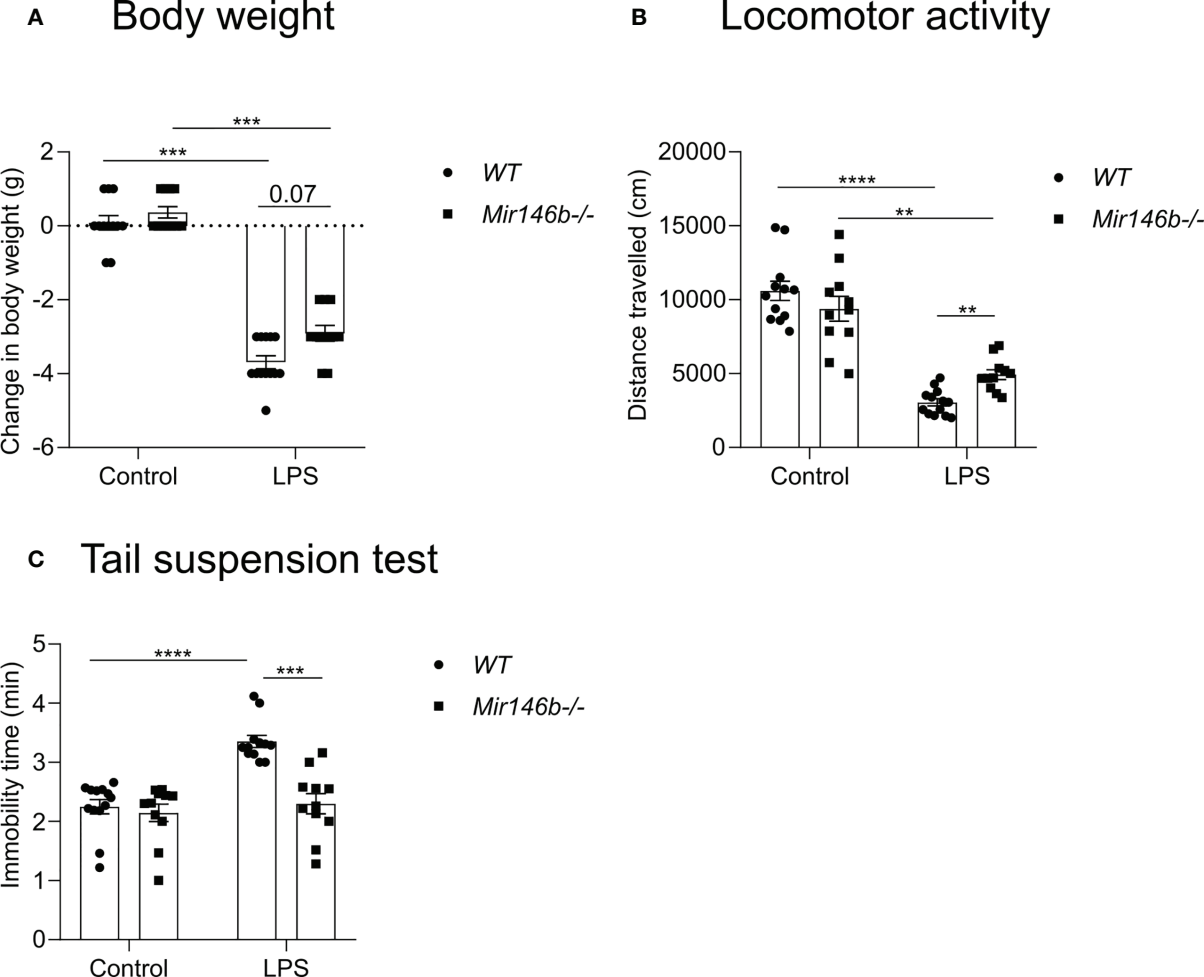
miR-146b deficient mice have reduced sickness behavior upon LPS challenge. Mice were injected with LPS in saline or equal volume of saline (control) and subjected to sickness behavior tests. **(A)** Change in body weight, **(B)** Locomotor activity and **(C)** Immobility time in tail suspension test was measured. Number of animals = 11-12. Data are represented as mean ± SEM; **p<0.01;***p<0.001; **** p < 0.0001 (Welch’s ANOVA with Dunnett’s multiple comparisons test).

We first evaluated changes in body weight before and 24 h after a high dose administration of LPS (1.5 mg/kg) and observed that LPS induced a decrease in body weight in both *WT* and *Mir146b-/-* mice (Welch’s ANOVA (F(3,40.12) = 120.3, p < 0.0001). Dunnett’s test revealed a significant effect (p < 0.001) of LPS treatment in both *WT* and *Mir146b-/-* mice. However, LPS-induced body weight loss was slightly less prominent in *Mir146b-/-* mice than that observed in *WT* mice (p = 0.07) (**Figure 3A**).

Next, reductions in locomotor activity were observed in both *WT* and *Mir146b-/-* mice after LPS treatment (Welch’s ANOVA F(3,21.5) = 50.01, p < 0.0001). Dunnett’s test demonstrated a significant reduction in locomotor activity in WT (p < 0.0001) and in *Mir146b-/-* mice (p < 0.001), whereas *Mir146b-/-* mice demonstrated a significantly (p < 0.01) less reduction in locomotor activity upon LPS challenge as compared with *WT* mice (**Figure 3B**).

Furthermore, we evaluated the development of depression-like behavior as evidenced by immobility time in the tail suspension test and observed increased immobility time upon LPS treatment (Welch’s ANOVA demonstrated a highly significant effect of LPS (F(3,23.1)=17.19, p < 0.0001). *Post hoc* analysis revealed a significant enhancement of immobility in *WT* mice upon LPS challenge (p < 0.0001). In contrast, no enhanced immobility was observed in *Mir146b-/-* mice compared to the untreated group (**Figure 3C**).

In conclusion, LPS-induced sickness behavior manifested by the rapid decrease in body weight, reduction in locomotor activity and development of depression-like behavior was less severe in *Mir146b-/-* mice as compared with *WT* littermates.

### LPS induced less activation in microglial morphology and Iba1 protein levels in *Mir146b-/-* mice

**Figure 4:**
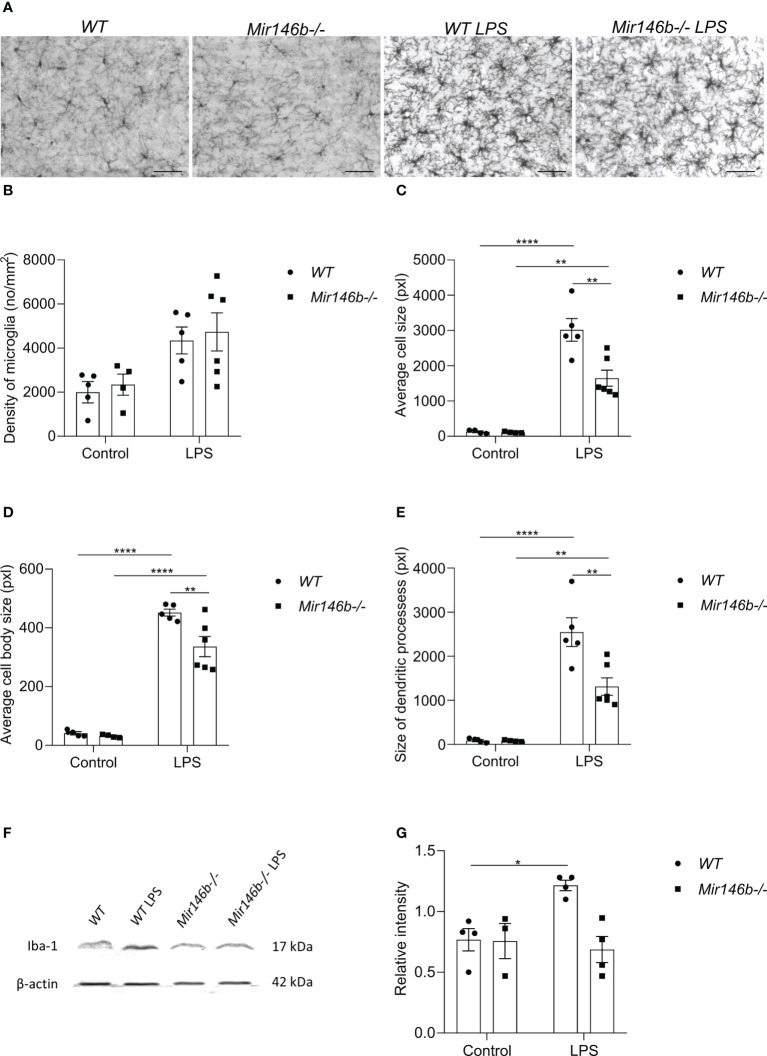
Microglial morphology and Iba1 protein levels upon LPS challenge in the hippocampus of miR-146b deficient and *WT* mice. Mice were injected with LPS in saline or equal volume of saline (control) followed by harvesting the tissues and immunohistochemistry. **(A)** Representative immunohistochemistry microphotographs of the hippocampal Iba1+ cells at 400× magnification. **(B)** Density of microglial cells, **(C)** Average cell size, **(D)** Average cell body size and **(E)** Size of dendritic processes of microglial cells in hippocampus of *WT* and *Mir146b-/-* mice upon LPS treatment. Number of animals = 5-6. Scale bar = 50 μm. Data are represented as mean ± SEM; ** p < 0.01, **** p < 0.0001 (Two-way ANOVA with Tukey’s multiple comparison test). For western blot, hippocampal lysates from LPS or saline injected *WT* and *Mir146b-/-* mice were probed for Iba1 protein. **(F)** Representative immunoblot for Iba1 protein, **(G)** Densitometry quantification of Iba1. Number of animals = 4. Data are represented as mean ± SEM; * p<0.05 (Welch’s ANOVA with Dunnett’s multiple comparisons test).

LPS is known to act through the innate immune toll-like 4 (TLR4) to induce an innate immune response, which causes, among other symptoms, sickness behavior. TLR4 is highly expressed in brain microglia, and excessive inflammation resulting from activation of this pathway in the brain has been implicated in various brain pathologies (40). Therefore, we next evaluated LPS-induced microglial activation by assessing their profiles of morphological changes. To do that, we stained the brain sections from WT and Mir146b-/- mice with antibody against Iba1, a marker for microglia (**Figure 4A**) and assessed microglial morphometrics, such as average cell size, cell body size and size of dendritic processes.

Our results demonstrated that LPS administration induced trends of increase in microglial density in both *WT* and *Mir146b-/-* mice (**Figure 4B**). Significant effect of LPS treatment (F(1,17) = 13.18, p = 0.0021) but not genotype and interaction were observed.

Next, we assessed microglial cell size (**Figure 4C**), cell body size (**Figure 4D**), and size of the dendritic processes (**Figure 4E**) separately. We detected increased values of all these parameters upon LPS challenge in both *WT* and *Mir146b-/-* mice, but which were less pronounced in *Mir146b-/-* mice compared to *WT* group. For average cell size, significant effects existed for LPS treatment (F(1,17) = 119.3, p < 0.0001), genotype (F(1,17) = 11.60, p = 0.0034), and interaction (F(1,17) = 11.25, p = 0.0038). For average cell body size, significant effects of LPS treatment (F(1,17) = 296.1, p < 0.0001),genotype (F(1,17) = 8.795, p = 0.0087), and interaction (F(1,17) = 6.604, p = 0.0199) also existed. For size of the dendritic processes, effects of LPS treatment (F(1,17) = 91.33, p < 0.0001), genotype (F(1,17) = 10.27, p = 0.0052), and interaction (F(1, 17) = 10.20, p = 0.0052) were significant as well.

In addition, we measured LPS-induced changes in Iba1 protein levels in the hippocampus of *Mir146b-/-* and *WT* littermates (**Figures 4F, G**). For Iba1 protein levels Welch’s ANOVA demonstrated significant effect (F(3,5.1) = 10.58, p = 0.028). *Post hoc* analysis demonstrated a significant increase in Iba1 protein upon LPS challenge in WT animals, but no significant changes were observed in *Mir146b-/-* mice (**Figure 4G**).

These results together demonstrated that LPS-induced activation of microglia, manifested by increased cell size, cell body size and size of the dendritic processes, was less pronounced in *Mir146b-/-* mice as compared to *WT* mice.

### LPS induced changes in microglial profile in *Mir146b-/-* mice

**Figure 5:**
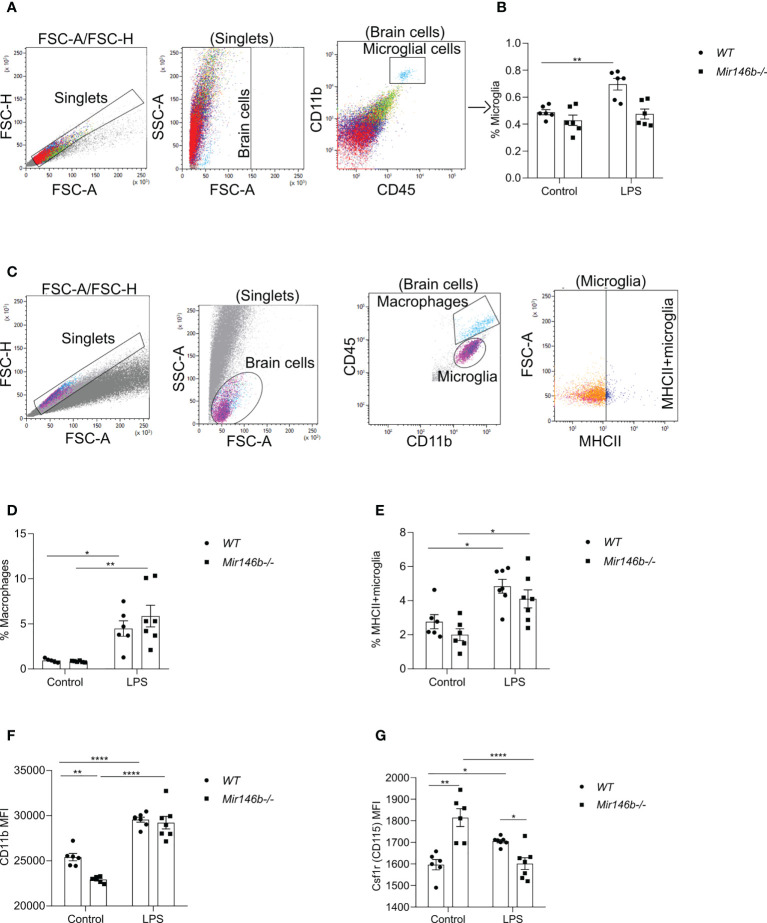
LPS-induced hippocampal microglial changes in *WT* and miR-146b deficient mice. Flow cytometry analysis with indicated markers was performed using brain cells from mice were injected with LPS in saline or equal volume of saline (control). **(A)** Representative gating strategy for quantification of microglial percentage. **(B)** Percentage of microglial cells among total brain cells. **(C)** Representative gating strategy for quantification of macrophages and MHCII+ microglia. **(D)** Percentage of macrophages **(E)** Percentage of MHCII+ microglia among microglial cells. **(F, G)** Mean fluorescent intensity (MFI) of CD11b and Csf1r (CD115) among microglial cells. Number of animals = 6-7. Data are represented as mean ± SEM; * p < 0.05, ** p < 0.01, **** p < 0.0001 (Two-way ANOVA with Tukey’s multiple comparison test).

We next tested whether systemic administration of LPS induces a different microglial profile in *WT* and *Mir146b-/-* mice using flow cytometry of hippocampus tissue 24 h after LPS or saline administration. A combination of cell-specific markers was used to identify CD11b+CD45 intermediate as microglia and CD11b+CD45 high as macrophages and repesentative isotypic controls are shown in (**Supplement Figure 2B**). Representative gating strategy for quantification of microglial percentage is shown in (**Figure 5A**) and we found increased percentage of microglia (p = 0.0027) upon LPS activation in *WT* mice, but not in *Mir146b-/-* mice (**Figure 5B**), with significant effect of LPS treatment (F(1,20) = 12.81, p = 0.0019), genotype (F(1,20) = 15.63, p = 0.0008) and interaction (F(1,20) = 5.150, p = 0.0345) being observed. The percentage of macrophages were elevated in the hippocampus in both *WT* (p = 0.0430) and *Mir146b-/-* mice (p = 0.0013) after LPS administration (**Figures 5C, D**) too, with significance in the effect of LPS treatment (F(1,20) = 26.22, p < 0.0001) but not genotype and interaction.

To assess the activation level of microglial cells after LPS stimulation, percentage of major histocompatibility complex II (MHC-II)-positive M1-type of pro-inflammatory microglia and mean fluorescence intensity (MFI) level of CD11b were measured. CD11b is constitutively expressed by microglia, but its expression is increased upon activation (41–43). The percentage of MHCII expressing microglia were significantly higher in *WT* LPS group (p = 0.0142) and *Mir146b-/-* LPS (p = 0.0131) compared to their respective controls (**Figure 5E**). Significance of LPS treatment (F(1,22) = 22.69, p < 0.0001) but not genotype and interaction were observed. As expected, the MFI level of CD11b was significantly higher after administration of LPS in both *WT* (p < 0.0001) and *Mir146b-/-* mice (p < 0.0001). No significant difference between the *WT* LPS and *Mir146b-/-* LPS groups was detected. However, the basal level of CD11b was lower in *Mir146b-/-* mice compared to *WT* mice (p = 0.0056) (**Figure 5F**), indicating reduced basal activation of *Mir146b-/-* microglia. Statistical analysis revealed significant effect of LPS treatment (F(1,22) = 132.2, p < 0.0001), genotype (F(1,22) = 9.717, p = 0.0050), and interaction (F(1,22) = 5.700, p = 0.0260). Altogether, these results suggest that microglial cells in *Mir146b-/-* mice showed less activation as compared to *WT* mice.

We also investigated the expression of macrophage colony-stimulating factor 1 receptor, Csf1r (CD115) on microglial surface. CSF1R signaling is essential for microglial survival, proliferation, and differentiation (44–47). Altered expression of CSF1R in microglia have been identified in many neuroinflammatory diseases (48, 49). An *in vivo* study had shown that inhibition of CSF1R attenuated neuroinflammation and reduced microglial proliferation in a murine acute LPS model (50). We found that LPS induced an increase in (p = 0.0411) Csf1r expression in the hippocampus of *WT* mice but decreased its expression in *Mir146b-/-* mice (p < 0.0001). Notably, the basal level of Csf1r was higher in *Mir146b-/-* mice compared to *WT* mice (p < 0.0001) (**Figure 5G**). Statistical analysis showed significant effects of genotype (F(1,22) = 4.535, p = 0.0446) and interaction (F(1,22) = 36.05, p < 0.0001) but not LPS treatment. Altogether, flow cytometry analysis results indicate that microglia of miR-146b deficient mice were less activated and more resistant to LPS treatment.

### LPS induced changes in microglia-mediated phagocytic activity in *Mir146b-/-* mice

**Figure 6:**
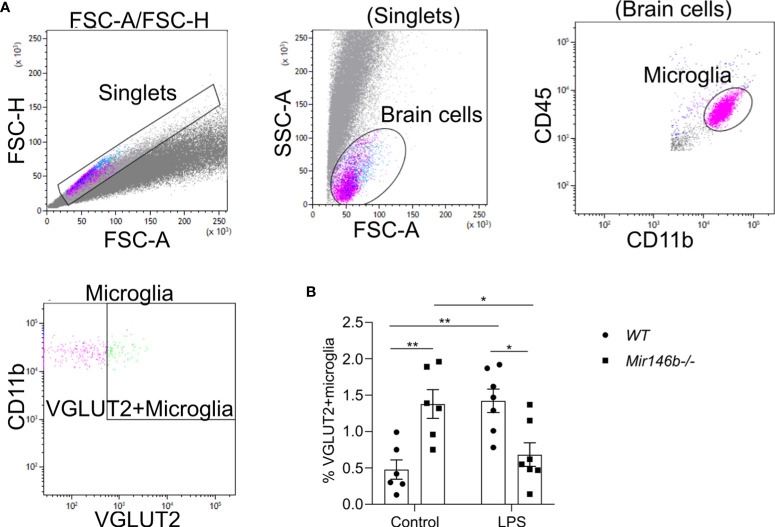
Phagocytic activity of hippocampal microglia in LPS-treated *WT* and *Mir146b-/-* mice. **(A)** Gating strategy for VGLUT2+ microglia among CD11b+CD45intermediate microglial cells. **(B)** Percentage of VGLUT2+ microglial cells upon LPS challenge in *WT* and *Mir146b-/-* mice. Number of animals = 6-7. Data represented as mean ± SEM; * p < 0.05, ** p < 0.01 (Two-way ANOVA with Tukey’s multiple comparisons test).

LPS-induced neuroinflammation has been linked to enhanced phagocytosis (51). Phagocytosis is an event, which has been associated with an anti-inflammatory profile of activated microglia (52). Therefore, we evaluated the microglial phagocytic activity upon LPS challenge in *Mir146b-/-* mice using intracellular staining Vglut2, a glutamatergic pre-synapse-specific protein, by flow cytometry similarly as performed before in (34, 53). Gating strategy with representative dot plots are presented in (**Figure 6A**). Statistical analysis revealed that *Mir146b-/-* mice have significantly higher percentage of Vglut2+ microglia than *WT* mice (p = 0.0062) in basal conditions suggesting that miR-146b deficiency results in an increased phagocytic activity of microglia. Upon LPS treatment, we observed higher levels of Vglut2 engulfment in *WT* mice (p = 0.0029), whereas *Mir146b-/-* mice showed a decrease in percentage of Vglut2+ microglia (p = 0.0033) (**Figure 6B**) compared to their controls. Statistical analysis revealed no significant effect of LPS treatment (F(1,22) = 0.57, p = 0.46) and genotype (F(1,22) = 0.23, p = 0.63) and highly significant effect of interaction (LPS x genotype, F(1,22) 24.59, p < 0.0001). These results together suggest that miR-146b deficiency reduces microglial ability to be converted into phagocytic phenotype upon LPS treatment.

### LPS induced changes in cytokine gene expression in *Mir146b-/-* mice

**Figure 7:**
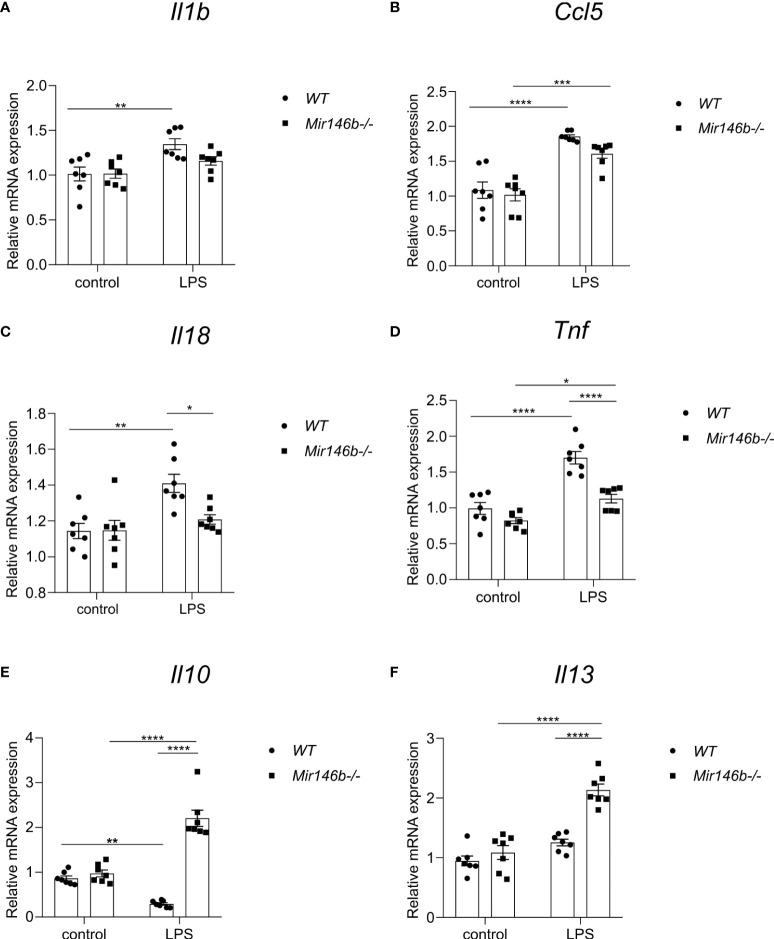
Gene expressional changes in the hippocampus of *Mir146b-/-* mice reveal reduced inflammatory response to LPS. Total RNA was purified from hippocampus of mice injected with LPS in saline or equal volume of saline (control). **(A)** Relative expression of *Il1b* **(B)** *Ccl5* **(C)** *Il18* **(D)** *Tnf* **(E)** *Il10* and **(F)** *Il13* was analyzed by RT-qPCR with primers for indicated genes. Number of animals = 7. Data represented as mean ± SEM; * p < 0.05, ** p < 0.01, **** p < 0.0001 (Data in panels **(A–C, E, F)** were analyzed using two-way ANOVA with Tukey’s multiple comparisons test; data in panel **(D)** did not show normal distribution and were transformed into logarithms (Y=log(Y) and further processed using one-way ANOVA with Tukey’s multiple comparisons test).

To answer the question whether reduced ability of LPS to induce neuroinflammation in *Mir146b-/-* mice was associated with changes in mediators of neuroinflammation, we evaluated mRNA expressions of genes encoding pro-inflammatory cytokines (*Il1b*, *Il18*, *Tnf, Il6, and ccl5*), anti-inflammatory cytokines (*Il10* and *Il13*), and NLRP3 inflammasome component (*Nlrp3)*. Under basal conditions, the mRNA expression levels of these genes did not significantly differ between *WT* and *Mir146b-/-* mice. However, the marked differences between genotypes appeared upon LPS administration (**Figure 7**).

LPS administration induced increased expression of *Il1b* and *Ccl5* mRNAs in *WT* mice, while in *Mir146b-/-* mice *Il1b* mRNA levels remained unchanged and *Ccl5* mRNA levels showed an increase in response to LPS (**Figures 7A, B**). A significant effect of LPS treatment on *Il1b* and *Ccl5* mRNA expression (F(1,24) = 15.40, p = 0.0006; F(1,24) = 69.70, p < 0.0001) was found.

Similarly, administration of LPS increased expression of *Il18* (p = 0.0019) and *Tnf* (p < 0.0001) mRNAs in *WT* mice, while *Il18* remained resistant (p = 0.7802) and *Tnf* was slightly increased (p = 0.0257) in *Mir146b-/-* mice upon LPS administration (**Figures 7C, D**). Significant effects of LPS treatment (F(1,24) = 12.96, p = 0.0014; F(1,24) = 51.85, p < 0.0001), genotype (F(1,24) = 4.759, p = 0.0392; F(1,24) = 27.64, p < 0.0001), and interaction (LPS x genotype, F(1,24) = 5.114, p = 0.0331; F(1,24) = 8.245, p = 0.0084) existed. Other pro-inflammatory cytokines *Il6* and inflammasome component *Nlrp3* also exhibited increased expression in *WT* mice upon LPS treatment, while in *Mir146b-/-* mice *Il6* and *Nlrp3* mRNA levels remained unchanged (**Supplement Figure 3**). Significant effects of LPS treatment was seen in both the mRNAs (F(1,24) = 18.54, p = 0.0002; F(1,24) = 29.24, p < 0.0001), but not genotype on both mRNAs, while for *Nlrp3*, a significant effect of interaction (F(1,24) = 20.63, p = 0.0001) was observed.

In *WT* mice, LPS administration reduced the level of anti-inflammatory cytokine *Il10* (p = 0.0036) but not *Il13*. Interestingly, in *Mir146b-/-* mice, the expressions of both cytokines were significantly increased (**Figures 7E, F**). Significant effects of LPS treatment (F(1,24) = 10.09, p = 0.0041; F(1,24) = 56.31, p < 0.0001), genotype (F(1,24) = 94.77, p < 0.0001; F(1,24) = 31.59, p < 0.0001), and interaction (LPS x genotype, F(1,24) = 75.30, p < 0.0001; F(1,24) = 16.68, p = 0.0004) were seen. Overall, miR-146b deficient mice had reduced levels of expression of pro-inflammatory cytokine genes and enhanced expression of anti-inflammatory genes upon LPS treatment compared with *WT* mice.

### miR-146a is upregulated in the hippocampus and microglial cells isolated from miR-146b deficient mice

**Figure 8:**
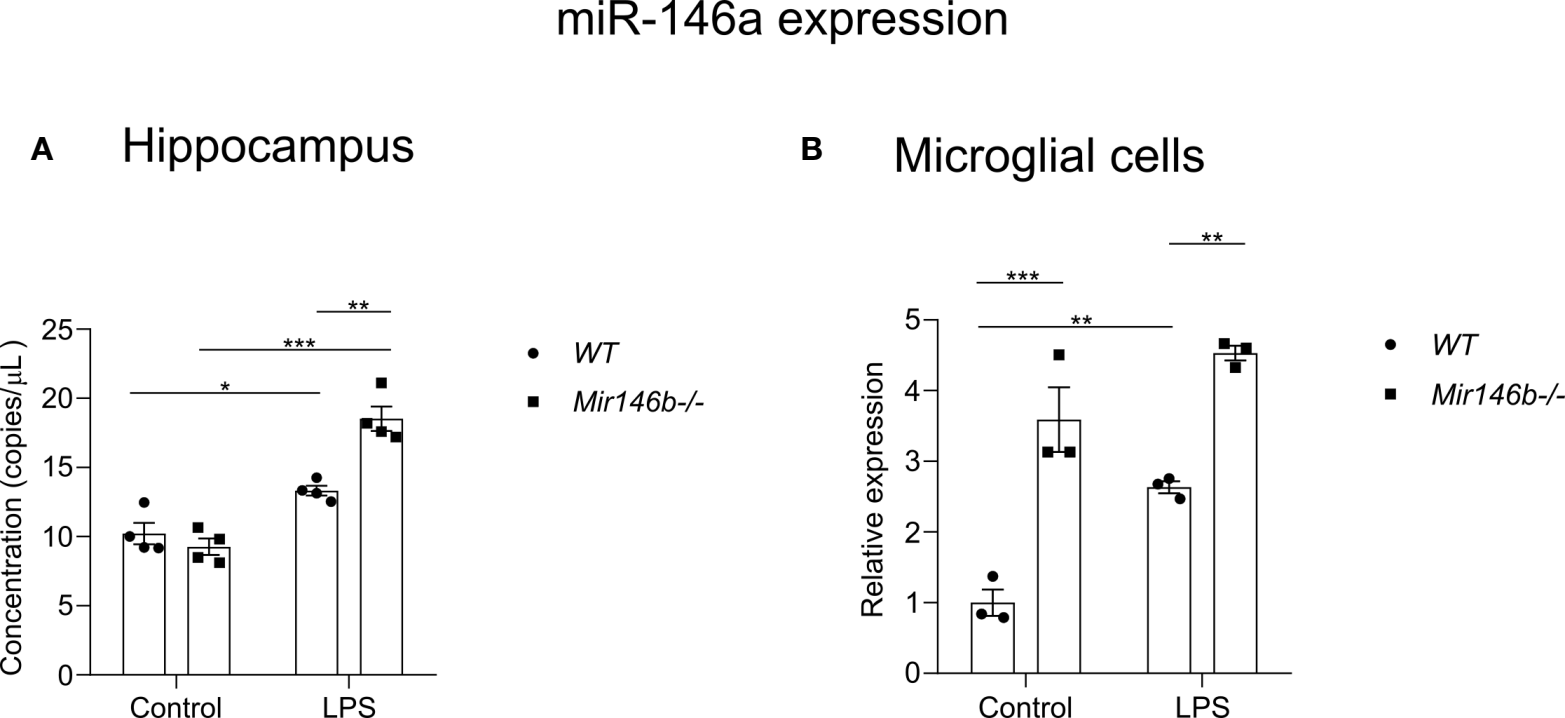
Basal and LPS-induced miR-146a expression in the hippocampal tissues and microglia of *Mir146b-/-* mice and *WT* littermates. **(A)** miR-146a expression in hippocampal tissues **(B)** miR-146a expression in microglial cells. Number of animals = 4 **(A)** and 3 **(B)**. * p=0.05, ** p<0.01, *** p<0.001. (Data in panel **(A)** were analyzed using two-way ANOVA with Tukey’s multiple comparisons test; data in panel **(B)** was analyzed using Welch’s ANOVA with Dunnett’s multiple comparisons test).

To answer the question of what mechanisms might be responsible for the observed attenuation of LPS-induced neuroinflammation in *Mir146b-/-* mice we measured the expression of miR-146a in these animals. First, we measured absolute miR-146a levels in the hippocampal tissues and secondly, we measured relative expression of miR-146a in microglial cells isolated from whole brain using percoll gradient. Experiments demonstrated significantly higher expression of miR-146a upon LPS challenge in the hippocampal tissue of *Mir146b-/-* mice (p < 0.0001) as compared to *WT* animals (p = 0.0016) (**Figure 8A**). A significant effect of LPS treatment (F(1,12) = 106.0, p < 0.0001) but not genotype and interaction (LPS x genotype, F(1,12) = 10.74, p = 0.0066) was found. In isolated microglial cells, both *WT* and *Mir146b-/-* mice showed overexpression of miR-146a upon LPS administration. Welch’s ANOVA of miR-146a expression in microglial cells also demonstrated a significant effect on LPS (F(3,4.2) = 86.89, p = 0.0003), whereas *post hoc* analysis showed revealed much higher increase (p = 0.0006) in miR-146a upon LPS challenge in *Mir146b-/-* mice as compared with *WT* mice. It should be also noted that basal miR-146a expression in *Mir146b-/-* mice was also significantly (p = 0.001) higher than in *WT* mice (**Figure 8B**).

### LPS-induced neuroinflammatory response is restored in double knockout *Mir-146a/b-/-* mice

**Figure 9:**
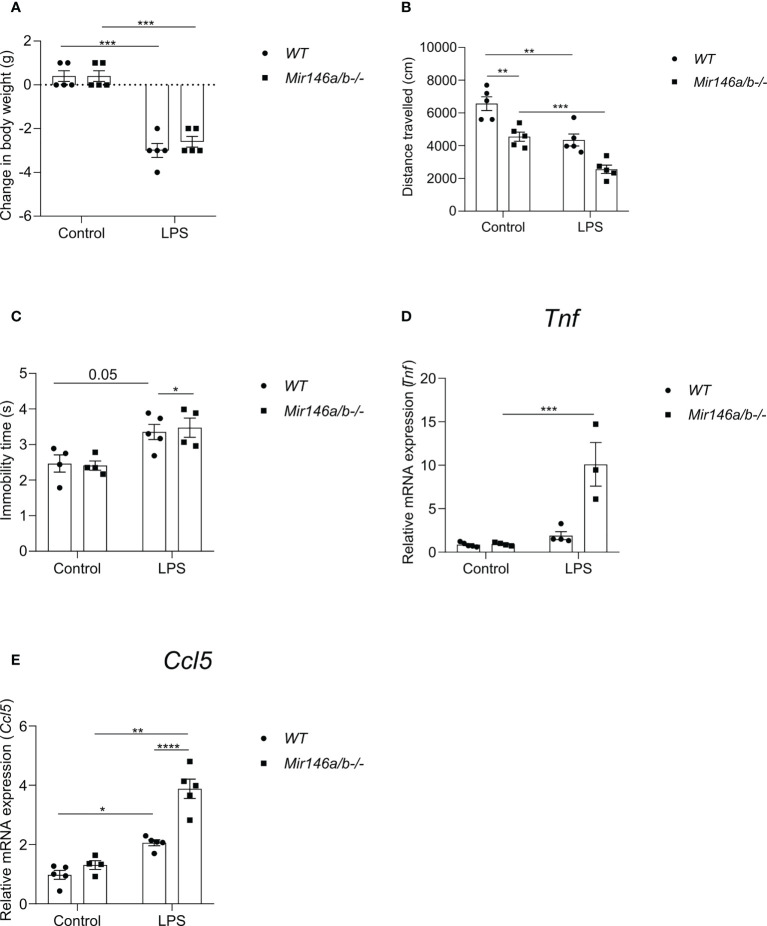
Effects of LPS on sickness behavior and proinflammatory cytokines in Mir146a/b-/- mice. **(A)** Change in body weight, **(B)** Locomotor activity and **(C)** Immobility time in tail suspension test was measured. **(D)** Relative expression of Tnf and **(E)** Ccl5 was analyzed by qPCR. Number of animals = 3-5. Data represented as mean ± SEM; * p < 0.05, ** p < 0.01, *** p < 0.001, **** p < 0.0001. (Welch’s ANOVA with Dunnett’s multiple comparisons test).

To evaluate whether miR-146a upregulation is responsible for the observed attenuation of neuroinflammation in *Mir146b-/-* mice, additional experiments were performed on *Mir-146a/b-/-* mice. We assessed LPS-induced sickness behavior in these animals and measured the mRNA expression of pro-inflammatory cytokines *Ccl5* and *Tnf* in the hippocampal tissues. In *Mir-146a/b-/-* mice, LPS administration induced sickness behavior similar to *WT* mice. For body weight changes, Welch’s ANOVA showed significant effect LPS (F(3,15.3) = 76.2, p < 0.0001), whereas *post hoc* analysis showed a similar decrease in body weight in both *WT* and *Mir-146a/b-/-* mice (**Figure 9A**). For locomotor activity Welch’s ANOVA also showed a significance (F(3, 8.759 = 21.26, p = 0.002) and *post hoc* analysis demonstrated significant decrease in locomotion in *WT* (p < 0.05) and *Mir-146a/b-/-*mice (p = 0.01) (**Figure 9B**). Immobility time in tail suspension test was significantly (p = 0.05) increased in *Mir-146a/b-/-* mice and a trend toward increase was also seen in *WT* animals (**Figure 9C**). The lack of significance in *WT* animals is probably due to the insufficient number of animals in a group. Further gene expression analysis in *Mir-146a/b-/-* mice showed an increased expression of pro-inflammatory cytokines upon LPS challenge (**Figures 9D, E**). For *Tnf* expression, Welch’s ANOVA showed significant effect (F(3,5.61) = 5.1 p = 0.047) and Dunnett’s test showed a significant effect of LPS treatment in *Mir-146a/b-/-* mice. For *Ccl5* expression, Welch’s ANOVA also demonstrated significant effect of LPS (F(3, 14.4) = 29.5, p < 0.0001) and Dunnetts’s test showed a significant increase in both *WT* (p < 0.001) and *Mir-146a/b-/-* mice (p = 0.04). These data show that LPS induces neuroinflammatory response in double knockout *Mir-146a/b-/-* mice similar to those observed in *WT* mice.

### LPS induced NF-κB activation, *Irak1* expression and interferon regulatory factor 7 (IRF7) expression in *Mir146b-/-* mice

To explore further mechanisms of the observed LPS-induced upregulation of miR-146a in *Mir146b-/-* mice, we focused on the transcriptional regulators of miR-146a expression NF-κB and *Irf7/3*.

**Figure 10:**
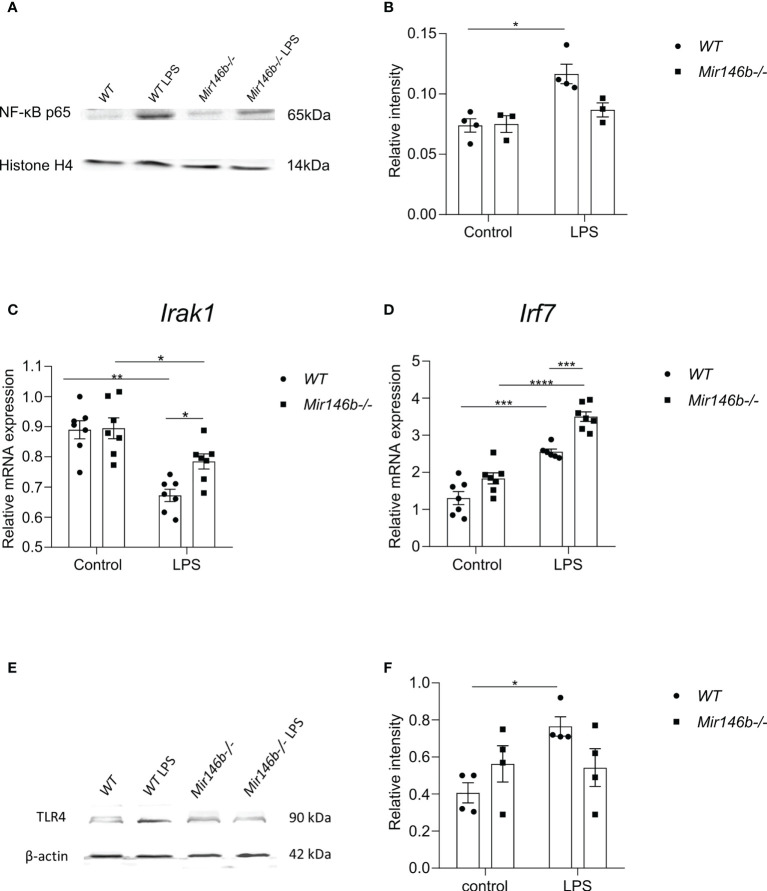
Effects of LPS on the activation of NF-κB, *Irak1* mRNA, miR-146a-transcriptional regulator *Irf7* and TLR4 protein expression levels in *Mir146b-/-* mice. **(A)** Representative immunoblot of hippocampal lysates probed for NF-κB p65 and histone H4 in the nuclear fractions of saline and LPS treated *WT* and *Mir146b-/-* mice. **(B)** Densitometry quantification of NF-κB p65 in the nuclear fractions was performed **(C)** Relative expression *Irak1* mRNA levels in qPCR **(D)** Relative expression of *Irf7* **(E)** Representative immunoblot probed for TLR4 and βactin and **(F)** Densitometry quantification of TLR4 in the hippocampal tissue isolated from saline and LPS treated *WT* and *Mir146b-/-* mice. Number of animals = 3-4 (Western blot) and 6-7 (qPCR). Data represented as mean ± SEM; * p < 0.05, ** p < 0.01, *** p < 0.001, **** p < 0.0001 (Two-way ANOVA with Tukey’s multiple comparison test).

As the expression levels of cytokines are NF-κB-dependent and NF-κB is the most important transcriptional regulator of miR-146a/b, we next assessed whether miR-146b deficiency may affect this pathway. The classical NF-κB pathway activation occurs *via* phosphorylation and following ubiquitination and degradation of IκB. The remaining NF-κB dimer (e.g., p65/p50 subunits) translocate to the nucleus, where it binds to the DNA consensus sequence of various target genes. Measuring p65 subunit in the nuclear fraction reflects the level of activation of the NF-κB pathway (54). Therefore, we next isolated the nuclear fraction of hippocampal cells and measured p65 and histone-4 levels with western blot. In *WT* mice, LPS treatment induced significant NF-kB activation (p = 0.039) as evidenced by the increased levels of p65 in the nuclear lysate. In contrast, no significant increase in p65 levels was observed in nuclear lysates purified from *Mir146b-/-* mice upon LPS challenge (**Figures 10A, B**) and the whole blot picture is provided in the (**Supplement Figure 4**). For p65 levels, two-way ANOVA demonstrated significant effect of LPS treatment (F(1,10) = 15.3; p = 0.0029) and significant effect of interaction between treatment and genotype (F(1,10) = 4.98; p = 0.05) but no effect of genotype.

We next analyzed mRNA expression of a miR-146a/b target, *Irak1* and TLR4 receptor protein levels in the hippocampus of *WT* and *Mir146b-/-* mice after LPS treatment. Interestingly, mRNA expression of *Irak1* was reduced in both *WT* and *Mir146b-/-* mice in response to LPS. However, the extent of *Irak1* reduction was more prominent in *WT* (p < 0.0001) than *Mir146b-/-* mice (p = 0.0478) (**Figure 10C**). Significant effects of LPS treatment (F(1, 24) = 34.30, P < 0.0001) and genotype (F(1, 24) = 4.421, P = 0.0462), but not interaction were observed. As previous data have demonstrated that upregulated miR-146a can negatively affect TLR4 expression (55–57), we analyzed TLR4 expression in miR-146b deficient mice upon LPS administration. Lysates of hippocampal tissues probed for TLR4 demonstrated a significant increase in TLR4 protein levels upon LPS administration in *WT* animals but not in *Mir146b-/-* mice (**Figures 10E, F**). These data suggest that miR-146a-mediated ability of targeting TLR4 might be responsible for the observed attenuation of NF-κB activation in *Mir146b-/-* mice.

Next, we explored a possible role of another group of transcriptional regulators *Irf3/7*. For *Irf3*, two-way ANOVA did not demonstrate significant effect. Multiple comparisons test revealed a small but significant increase in expression upon LPS treatment only in *Mir146b-/-* mice (**Supplement Figure 5**). For *Irf7*, two-way ANOVA demonstrated highly significant effect of LPS treatment: (F(2,23) = 82.4, p < 0001), significant effect of genotype: (F(2,23) = 11.9, p = 0.002), and significant effect of interaction: (F(2,23) = p = 0.02). Multiple comparisons test showed a significant increase in *Irf7* expression upon LPS challenge in both *WT* (p < 0.001) and *Mir146b-/-* (p < 0.0001) mice (**Figure 10D**). Furthermore, LPS-induced changes in *Irf7* expression were significantly (p < 0.001) higher in *Mir146b-/-* mice as compared with their *WT* littermates.

These data suggest that *Irf7* is most probably responsible for the observed upregulation of miR-146a in *Mir146b-/-* mice.

## Discussion

Previous research has demonstrated that miR-146 family members act as negative modulators of inflammation (12, 28, 31). Several other studies have also demonstrated that deficiency in miR-146a results in an exaggerated inflammatory response to the LPS challenge (18, 19, 58), whereas miR-146a overexpression reduces microglia mediated neuroinflammatory responses and inhibits sickness behavior (59, 60). Paradoxically, our study revealed that *Mir146b-/-* mice demonstrated attenuated neuroinflammatory response upon LPS challenge as compared with their *WT* littermates. Reduced neuroinflammatory status of *Mir146b-/-* mice upon LPS challenge was evidenced by the reduced intensity of sickness behavior and demonstrated less morphological characteristics of microglial activation. Although sickness behavior is believed to be associated with neuroinflammation, the impact of peripheral inflammatory reaction cannot be excluded (61, 62). Concordantly, flow cytometry experiments demonstrated an increased percentage of microglia in *WT* mice but not in *Mir146b-/-* mice upon LPS administration. In addition, the activation marker for microglial CD11b was decreased at basal levels in *Mir146b-/-* mice indicating that miR-146b deficiency causes lesser microglial activation. Furthermore, LPS mediated increased phagocytosis capacity in *WT* microglia and but not in *Mir146b-/-* mice.

In line with behavior studies and microglial status, LPS-mediated stimulation of the expression of pro-inflammatory cytokines *Il1b*, *Ccl5*, *Tnf*, *Il18, Il6 and Nlrp3* mRNAs was weaker in *Mir146b-/-* LPS mice compared to *WT* LPS animals. These data indicate that miR-146b deficient mice are more immunotolerant to the LPS-induced inflammatory signal than their *WT* littermates.

One possible explanation for a seemingly paradoxical consequence of miR-146b deficiency may come from the observed over-expression of miR-146a in these animals when treated with LPS. The increase in expression of miR-146a upon LPS challenge was evident in hippocampal tissues as well as in microglia isolated from the hippocampal tissue. Although the only marginal increase in miR-146a copy number in these animals was observed, the relevance of miR-146a over-expression in *Mir146b-/-* mice was confirmed by the experiments conducted on double knockout mice deficient for both miR-146a and miR-146b. Indeed, in these animals, LPS-induced sickness behavior and expression of pro-inflammatory cytokines were similar or even higher than those observed in *WT* animals.

It seems that blunted inflammatory response upon LPS challenge observed in *Mir146b-/-* mice is due to the reduced activation of NF-κB. The mechanisms of miR-146a-mediated reduction of neuroinflammatory response remains obscure. It seems unlikely that major regulatory activator of NF-κB, *Irak1* is implicated in the observed reduced activity of NF-κB as reduction in the expression of *Irak1* was higher in *Mir146b-/-* mice as compared with *WT* animals. More likely, reduced activation of NF-κB is due to the diminished ability of TLR4 to become upregulated in response to LPS. Indeed, several studies have demonstrated upregulation of TLR4 protein upon LPS challenge (63, 64). In our experiments, similar upregulation was also observed in *WT* animals and in contrast, *Mir146b-/-* mice TLR4 protein remained unchanged after LPS administration. It is known that TLR4 is a target of miR-146a (55–57) and thereby causing decreased activation of NF-κB and reduced inflammation.

**Figure 11:**
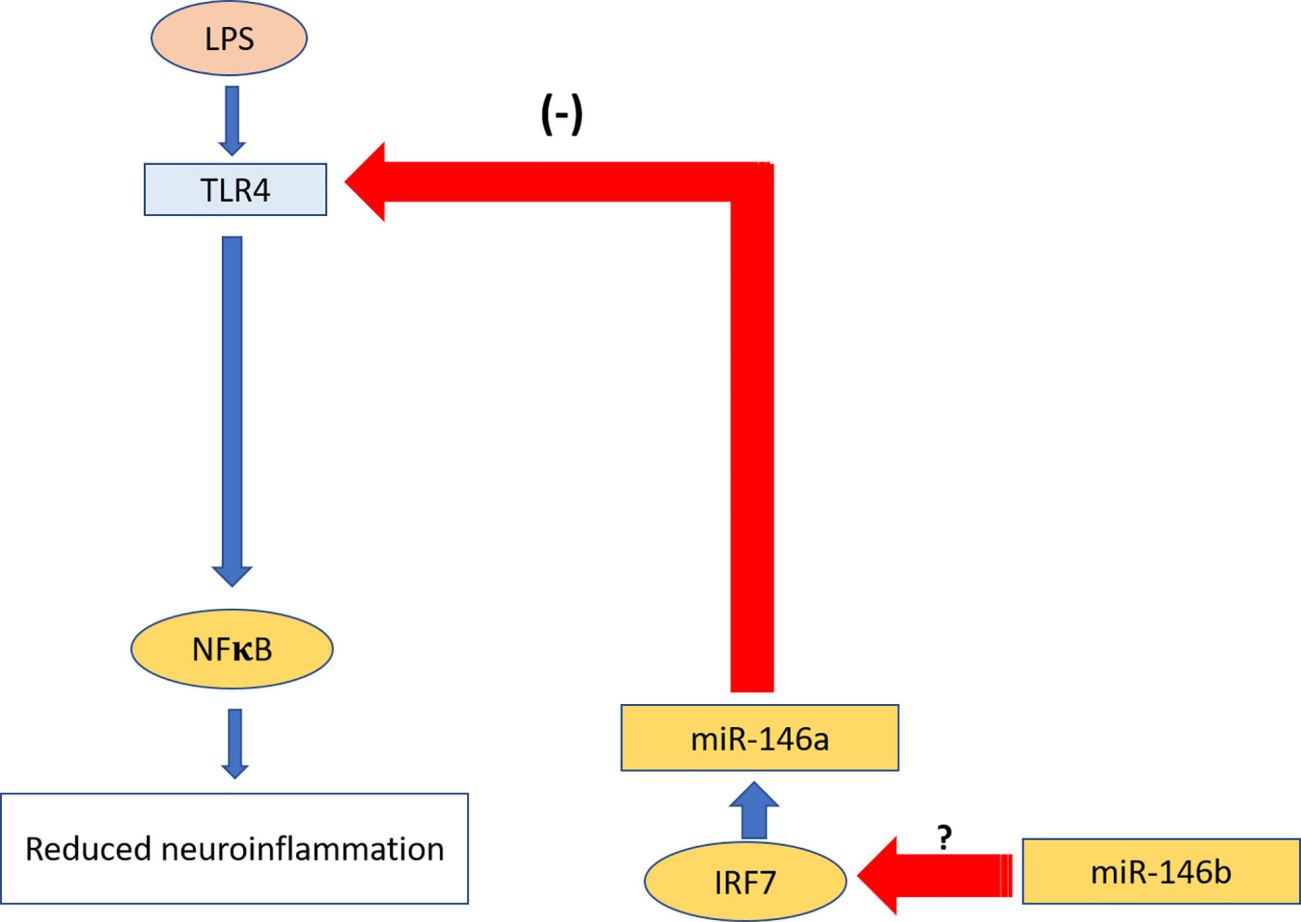
Summary of the crosstalk between miR-146a and miR.146b in the regulation of neuroinflammation. miR-146b leads to over-expression of miR-146a transcriptional regulator *Irf7*, which in turn induces upregulation of miR-146a. miR-146a inhibits activation of TLR4 upon LPS challenge and consequent inhibition of neuroinflammation. Red arrows show inhibition; blue arrows show activation.

To explain possible mechanisms of the observed miR-146a upregulation we next searched for transcription regulators such as interferon regulatory factor 3/7 (*Irf3/7*) responsible for miR-146a overexpression. There is evidence that *Irf3/7* plays an essential role in the LPS-induced interferon-β gene expression and endotoxic shock (65) and overexpression of *Irf3/7* suppressed inflammatory reaction upon LPS challenge (66). Indeed, our experiments demonstrated that *Irf7* and, to a lesser extent, *Irf3* become highly upregulated upon LPS challenge in miR-146b deficient mice. We, therefore, propose that upregulation of *Irf7* leads to the over-expression of miR-146a, which in turn inhibits NF-κB activation leading to the blunted inflammatory response. The crosstalk between miR-146a and miR-146b in the regulation of neuroinflammation is summarized in **Figure 11**.

It should be noted that the mechanism by which miR-146b can regulate the expression of *Irf3/7* remains unknown and needs to be studied in the future. In addition, a possible impact of other transcriptional regulators such as CCAAT-enhancer-binding-protein-β (C/EBPβ) in the observed overexpression of miR-146a in *Mir146b-/-* mice upon LPS treatment remains unknown. We also do not exclude the impact of systemic inflammation and peripheral miR-146b deficiency in the observed blunted neuroinflammatory response in miR-146b deficient mice. It is not excluded that miR-146b deficiency can promote invasion of the peripheral immune cells expressing miR-146a to the brain *via* disruption of the blood-brain-barrier (BBB). Wnt/β-catenin signaling is essential for maintaining the integrity of the adult BBB in physiological and pathological conditions (67) as its inhibition or deficiency causes BBB breakdown. Previous studies have shown that miR-146b induces silencing of Secreted frizzled-related protein 1 (SFRP1) which acts as an antagonist of Wnt signaling (68). Thus, miR-146b deficiency might induce indirect inhibition of Wnt/β-catenin signaling and weakness of BBB. This hypothesis, however, remains highly speculative as no direct studies on BBB permeability in miR146b deficient mice were conducted so far.

## Conclusion

In the present study, we utilized different methods to examine LPS-induced neuroinflammation in miR-146b deficient mice. It was found that LPS-induced neuroinflammation is attenuated in these animals and it was evidenced by the reduced signs of sickness behavior, and reduced activation of microglia. Gene and protein expression analysis revealed that LPS administration induced weaker upregulation of proinflammatory cytokines and NF-κB activation in the brain of *Mir146b-/-* mice compared to *WT* mice. The blunted neuroinflammatory reaction upon LPS challenge observed in *Mir146b-/-* mice is the most probably due to the compensatory over-expression of miR-146a. Over-expression of miR-146a is mediated by the induction of transcription regulator *Irf7* and, to a lesser extent, *Irf3* in the hippocampal tissue. Our data show the existence of crosstalk between miR-146a and miR146b in the regulation of neuroinflammation.

## Data availability statement

The original contributions presented in the study are included in the article/**Supplementary Material**. Further inquiries can be directed to the corresponding author.

## Ethics statement

The animal study was reviewed and approved by Animal Experimentation Committee at the Estonian Ministry of Agriculture (no. 183 and 158, 2021).

## Author contributions

KC: Designed and performed all experiments, writing - original draft, investigation, formal analysis. MJ: Performed behavioral animal experiments and perfusion and protein expression analysis. MG: Performed protein expression analysis and qPCR. LY: Performed behavior. TŽ: Performed Iba1 immunohistochemistry. MP: Quantification of miRNA. NM and MB: construction of mice line. AR: Writing - review and editing, investigation supervision, project administration, funding acquisition. LT: Writing - review & editing, conceptualization, supervision, project administration, funding acquisition. AZ: Writing – original draft, review & editing, conceptualization, supervision, project administration, funding acquisition. All authors contributed to the article and approved the submitted version.

## Funding

AZ is supported by the Estonian Research Council personal research funding team grant project No. PRG878. LT is supported by the Estonian Research Council-European Union Regional Developmental Fund Mobilitas Pluss Program No. MOBTT77 and AR is supported by European Regional Development Fund (Project No. 2014-2020.4.01.15-0012) and a personal grant from Estonian Research Council, PRG1259.

## Acknowledgments

The authors would like to thank Anu Remm for helping with genotyping of *Mir146b-/-* mice and Kai Kisand for providing histone antibody.

## Conflict of interest

The authors declare that the research was conducted in the absence of any commercial or financial relationships that could be construed as a potential conflict of interest.

## Publisher’s note

All claims expressed in this article are solely those of the authors and do not necessarily represent those of their affiliated organizations, or those of the publisher, the editors and the reviewers. Any product that may be evaluated in this article, or claim that may be made by its manufacturer, is not guaranteed or endorsed by the publisher.
