# Supplementary material for: Paradoxical attenuation of neuroinflammatory response upon LPS challenge in miR-146b deficient mice
Source: Front Immunol. 2022 Oct 31;13:996415. doi: 10.3389/fimmu.2022.996415 (PMC9659615; doi:10.3389/fimmu.2022.996415)
Supplement: Supplementary file 1 [file DataSheet_1.docx]

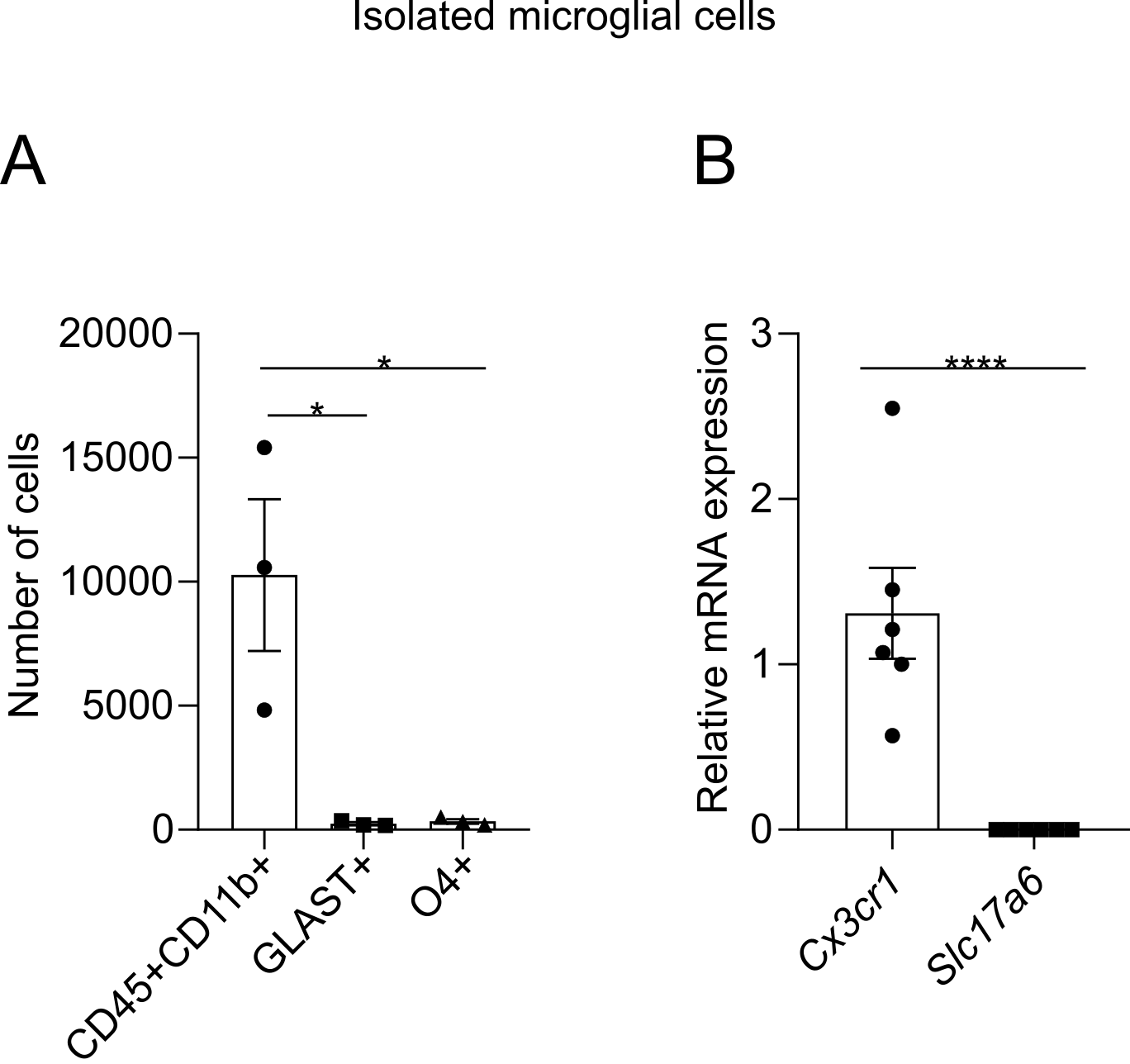


**Supplement Figure 1.** Characterization of isolated microglia cell fraction. The purity of isolated microglial cells was assessed by flow cytometry and RT-qPCR methods. (A) The cells were stained with cell surface makers for CD45+ CD11b+ for microglial cells, GLAST+ for astroglial cells and O4 for oligodendrocyte precursor cells and number of respective cells were measured by flow cytometry. (B) mRNA expression of *Cx3cr1* (microglial marker) and *Slc17a6* (neuronal marker) in isolated microglial cells. Number of animals = 3-6. Data represented as mean ± SEM; * p < 0.05, **** p < 0.0001 (Students t-test and Tukey’s multiple comparisons test).


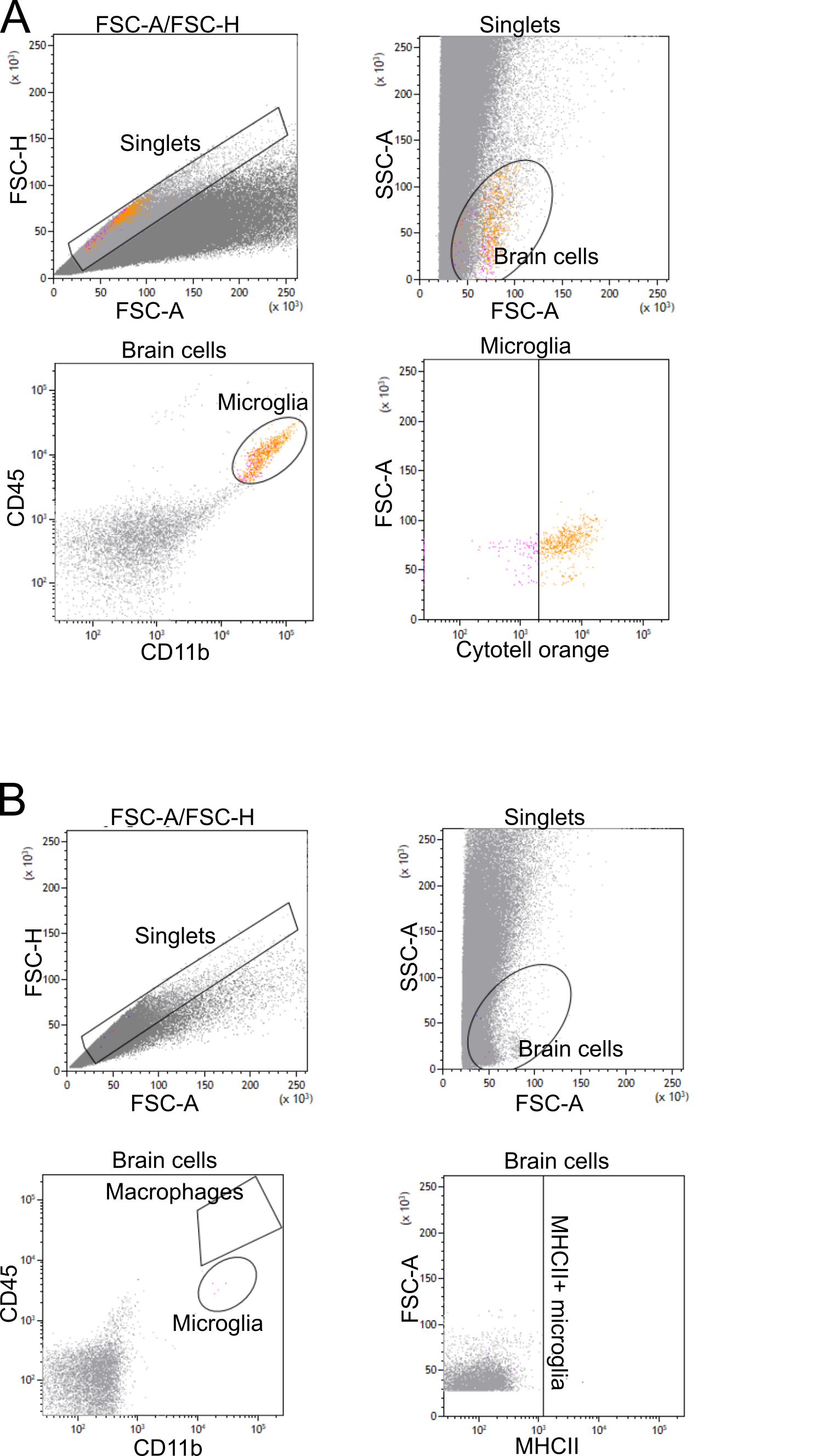


**Supplement Figure 2.** (A) Representative dot plots showing gating strategy of the cells stained with CytoTell orange (live cell staining); (B) Representative dot plots of isotypic controls used in flow cytometry staining.


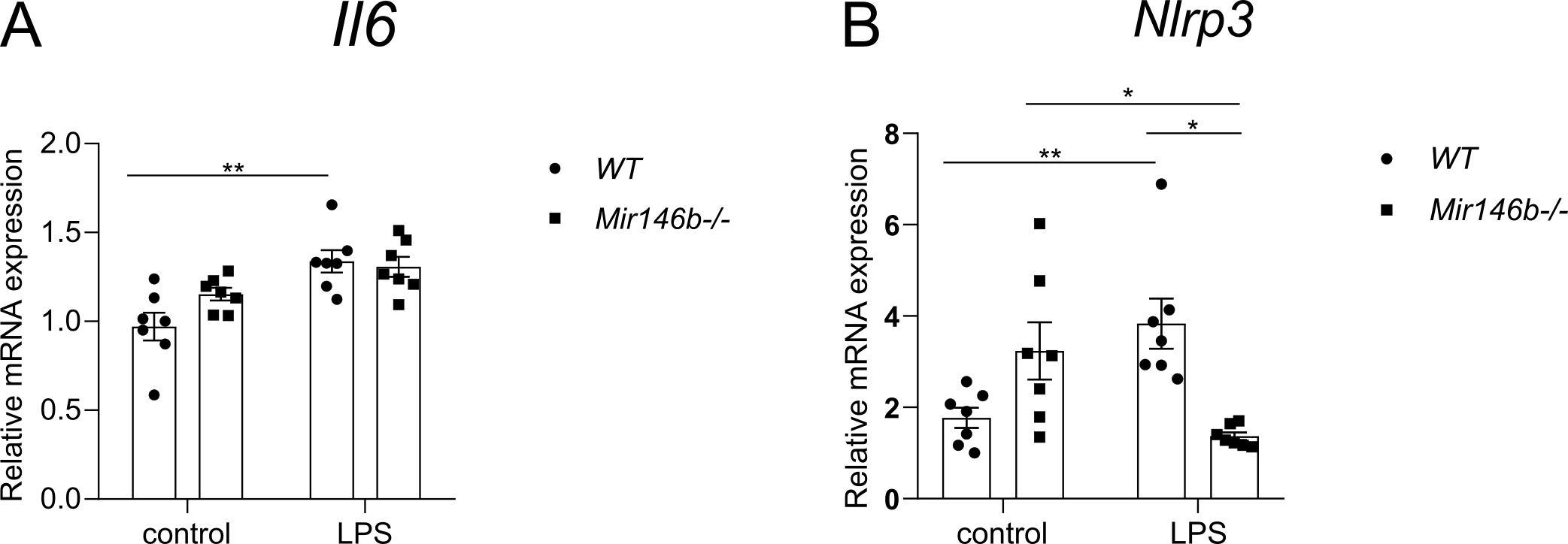


**Supplement Figure 3**. LPS-induced gene expression changes in the hippocampus of *WT* and *Mir146b-/-* mice. Relative mRNA expression levels of *Il6* (A) and (B) *Nlrp3* in *WT* and *Mir146b-/-* mice upon LPS administration. Number of animals = 7. Data represented as mean ± SEM; * p < 0.05, ** p < 0.01 (Tukey’s multiple comparisons test).


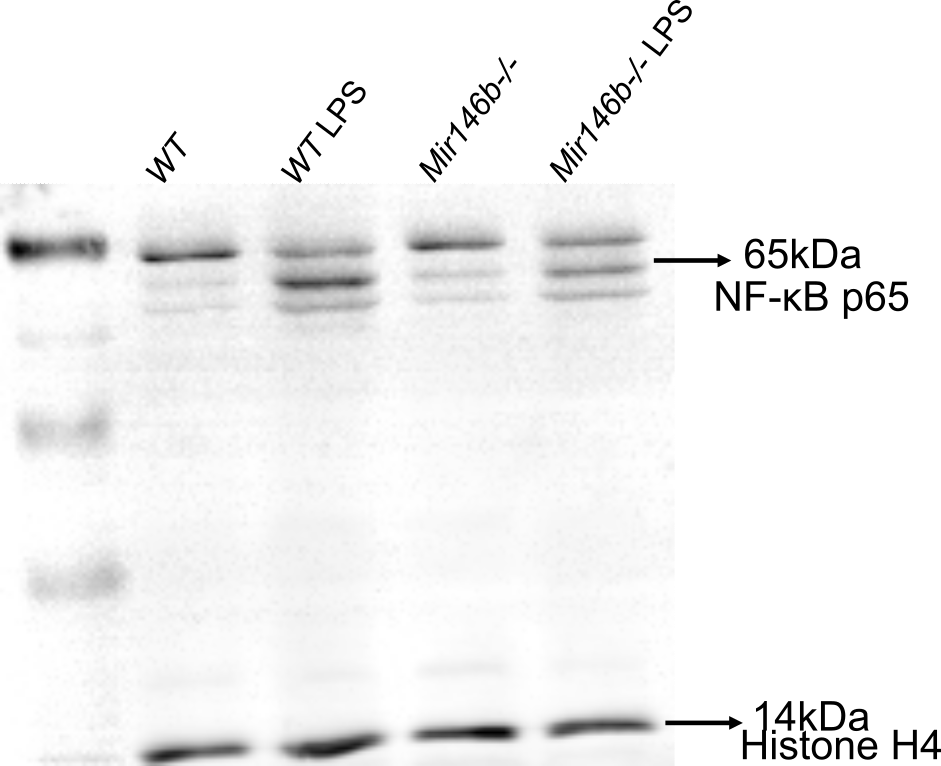


**Supplement Figure 4.** Representative original western blot showing LPS-induced NF-κB activation in *WT* and *Mir146b-/-* mice.

**Supplement Figure 5**. LPS-induced *Irf3* expression changes in the hippocampus of *WT* and *Mir146b-/-* mice. Relative mRNA expression levels of *Irf3* in *WT* and *Mir146b-/-* mice on LPS administration. Number of animals = 7. Data represented as mean ± SEM; * p < 0.05 (Tukey’s multiple comparisons test).

**Supplementary table 1. RT-qPCR Primers**

| **Gene name** | **Primer Sequence 5’-3’** | |
| --- | --- | --- |
| ***Cx3cr1*** | **Forward** | GAGTATGACGATTCTGCTG |
|  | **Reverse** | CAGACCGAACGTGAAGACG |
| ***Slc17a6*** | **Forward** | GCTGGAAAATCCCTCGGACAG |
|  | **Reverse** | TCGCATAGCGGAGCCTTCTT |
| ***Il1b*** | **Forward** | TGAAGAAGAGCCCATCCTCTG |
|  | **Reverse** | GGAGCCTGTAGTGCAGTTGT |
| ***Nlrp3*** | **Forward** | AGGCTGCTATCTGGAGGAACT |
|  | **Reverse** | CATCTTCAGCAGCAGCCCTT |
| ***Tnf*** | **Forward** | GTAGCCCACGTCGTAGCAAA |
|  | **Reverse** | TTGAGATCCATGCCGTTGGC |
| ***Il18*** | **Forward** | TCAAAGTGCCAGTGAACCCC |
|  | **Reverse** | GGTCACAGCCAGTCCTCTTAC |
| ***IL6*** | **Forward** | CTGCAAGAGACTTCCATCCAG |
|  | **Reverse** | AGTGGTATAGACAGGTCTGTTGG |
| ***Ccl5*** | **Forward** | GCCTCACCATATGGCTCGGACAC |
|  | **Reverse** | TTGACGTGGGCACGAGGCAG |
| ***Il10*** | **Forward** | GGCGCTGTCATCGATTTCTC |
|  | **Reverse** | ATGGCCTTGTAGACACCTTGG |
| ***Il13*** | **Forward** | GACCAGACTCCCCTGTGCAACG |
|  | **Reverse** | AGGGCTACACAGAACCCGCCA |
| ***Irak1*** | **Forward** | TGTGAGGACACAAGGTGCAA |
|  | **Reverse** | TAGGCTGGGTGCTTTTCAGG |
| ***Irf7*** | **Forward** | CGGGGACCTCTTGCTTCAG |
|  | **Reverse** | CAAGGCTGCGCTCAGGA |
| ***Irf3*** | **Forward** | ATTCCTCCCCTGGCTAGAGCAT |
|  | **Reverse** | CCTCGTTCGGCTCTCGTC |
| ***Gapdh*** | **Forward** | GTCATATTTCTCGTGGTTCACACC |
|  | **Reverse** | CTGAGTATGTCGTGGAGTCTACTG |
